# An anthropogenic model of cardiovascular system adaptation to the Earth’s gravity as the conceptual basis of pathological anthropology

**DOI:** 10.1186/s40101-021-00260-2

**Published:** 2021-08-27

**Authors:** G. S. Belkaniya, L. R. Dilenyan, D. G. Konkov, A. Wsol, A. K. Martusevich, L. G. Puchalska

**Affiliations:** 1 Laboratory of Medical Expert Systems “Anthropos Systems Lab.”, Vinnitsa, Ukraine; 2 Privolzhsky Research Medical University, Nizhny Novgorod, Russia; 3 National Pirogow Memorial Medical University, Vinnitsa, Ukraine; 4 Department of Experimental and Clinical Physiology, Laboratory of Centre for Preclinical Research, Medical University of Warsaw, ul. Banacha 1b, 02-097 Warsaw, Poland

**Keywords:** Earth’s gravity, Upright walking, Cardiovascular system, Influence of gravity, Blood circulation, Adaptation, Anthropogenic model, Physio-anthropology, Pathological anthropology

## Abstract

Applying human biological evolution to solve topical problems of medicine and preventive cardiology was inspired by the realization of the need for clinical and experimental studies of biological (evolutionary) prerequisites in the occurrence of a pathology. Although it has been stated that there is a need to provide a full biological understanding of features, including those that increase an animal’s vulnerability to diseases, unfortunately, in this regard, erectile and associated adaptations to the Earth’s gravity in their physiological and pathological manifestations have not been considered. At the same time, it should be noted that humans, unlike other animal species, have the greatest vulnerability of the cardiovascular system (CVS). The latter is associated with fundamental differences in the functioning and regulation of the CVS by the influence of gravity on blood circulation in humans as upright creatures. Based on a review of comparative physiological, ontogenetic, and clinical studies from an evolutionary perspective, the idea of adaptation to the Earth’s gravity when walking upright in humans is justified as an anthropogenic basis for the physiology and pathology of the cardiovascular system and hemodynamic support systems (physio-anthropology and pathological anthropology).

## Introduction

In the course of the unique biological experiment of evolution (the transition to orthograde positional statics and upright walking), the problem of adaptation to the sharply increased influence of the hydrostatic effect of the Earth’s gravity on blood circulation was solved by forming a number of structural and functional transformations in the cardiovascular system (CVS) [1–4], which determined the features of its reactivity to various effects in orthostatics [5–10]. At the same time, the general trend of all changes in the reactive properties of the cardiovascular system observed in phylogenesis and ontogenesis is to improve and strengthen the functioning of pressor regulation mechanisms [4, 11–15]. This is reflected in the increasing representation of CVS pressor reactions in higher mammals in contrast with the phylogenetically preceding animal species [11, 16], as well as in the prevalence of the pressor effect of changes in blood pressure under various influences, especially in humans [5, 8, 17–21]. This phylogenetic specialty is well-illustrated by the age-related dynamics of changes in blood pressure [11, 22–24]. These studies show that the greatest increase in blood pressure is detected at the initial stages of postnatal ontogenesis, when the child learns to walk upright. It is most noticeable in the first years of life, when the child gets on its feet and begins to walk. Further ontogenetic adaptation of the CVS to the gradually increasing hydrostatic effect in blood circulation is the parallel increase in body linear dimensions and blood mass as well as the lengthening of the active period of the day. This change is associated with the expansion of human activity in terms of vertical posture and leads to an increase in the pressor mechanisms of blood pressure regulation [4, 21, 25–28]. Successful modeling of arterial hypertension in monkeys with their semi-vertical statics with increased exposure to the influence of the hydrostatic effect of blood circulation in experimental bipedalism was not accidental [11, 17, 29]. The present review aims to exclude the problem of gravity-associated pathology based on the physio-anthropological approach.

## Physiological antropology of cardiovascular adaptation to the Earth’s gravity

**Fig. 1:**
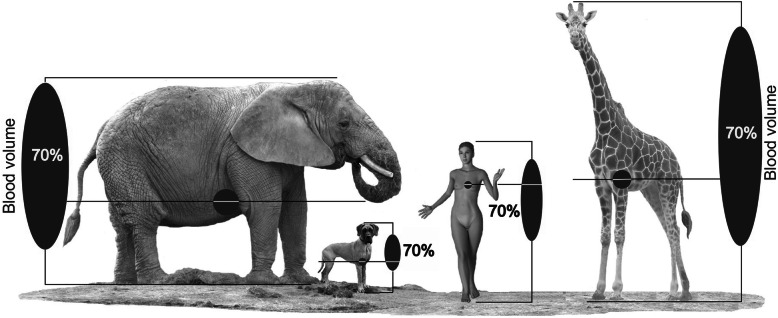
Distribution of blood volume and the ratio of the blood column relative to the heart level in humans (as an upright creature) and pronograde animals with four-legged locomotion

Based on the known dependence [30–33] of the value of the gravitational component (*E*—potential energy of gravity) of blood circulation (*E* = *g × m* × *h*, where *m* and *h* are the mass and height of the blood column below the heart level, respectively, and *g* is the gravitational constant), it is important to take into account that the influence of gravity (the hydrostatic effect) on blood circulation is progressively enhanced during the evolutionary development of animal organisms due to increasing linear body size and the transition from pronograde to semi-vertical and orthograde positional statics in primates as well as due to increasing linear body size and blood mass in postnatal ontogenesis. This effect reaches a comparative species maximum in an upright human (about 70% of the total blood volume in orthostatics is located below the heart level), whereas in animals with pronograde positional statics and quadrupedal locomotion, most of the intravascular blood volume is at the heart level and above [4, 11, 21, 32–34] (Fig. 1). This fundamentally distinguishes human blood circulation from all pronograde animals with four-legged locomotion, despite the common structure and functioning of the CVS in humans and in animals. It must be underlined that the formative influence of the Earth’s gravity and adaptation to it affected almost all of the systems of the human body, ensuring the proper functioning of the human body in its characteristic condition of upright walking [12, 32, 35, 36].

**Fig. 2:**
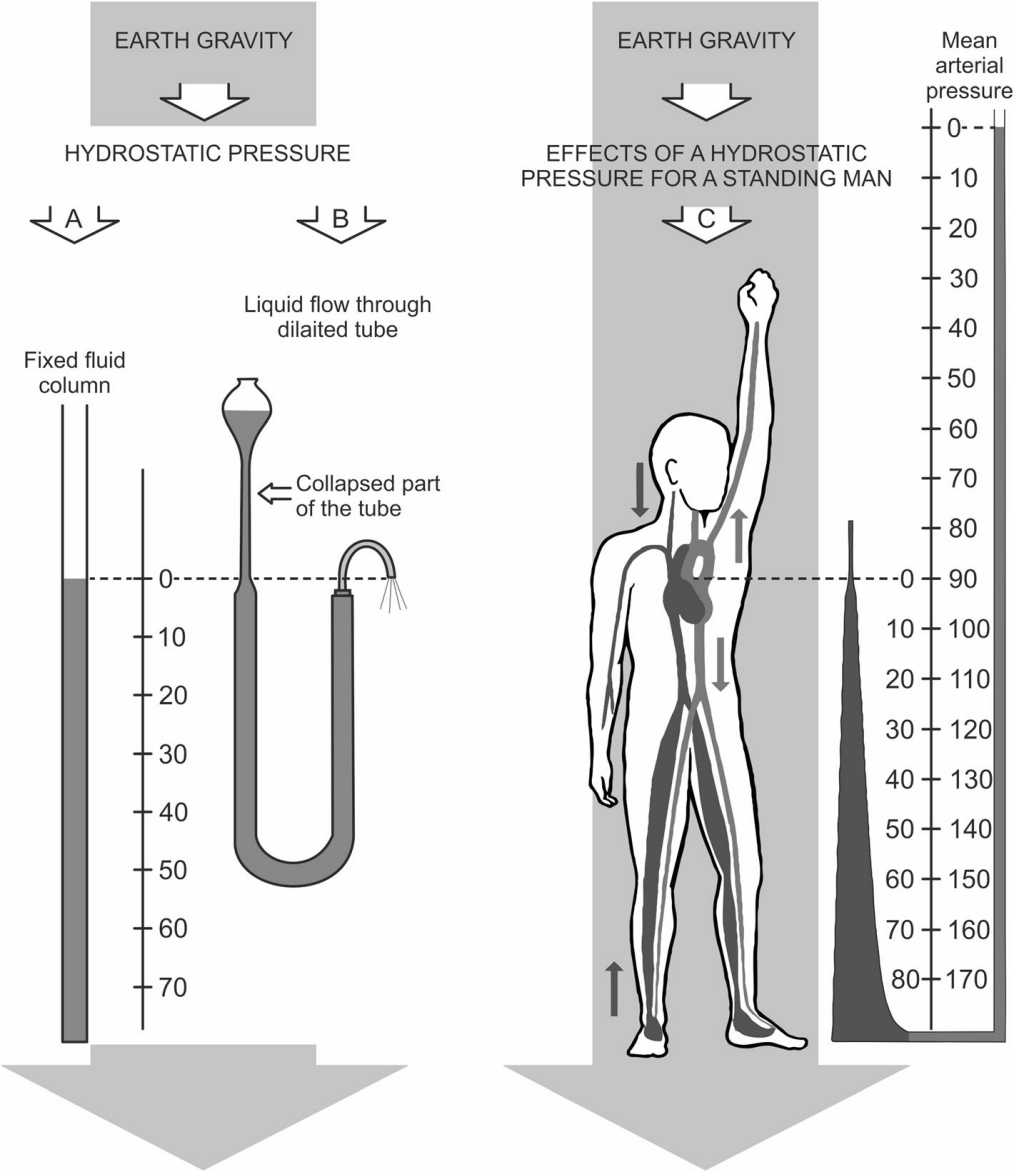
Physical nature and the effects of hydrostatic pressure according to Rushmer [33]. **A** The pressure of a column of liquid depends on gravity and the vertical distance from the pressure measurement point to the meniscus of the upper level of the liquid. **B** The fluid-filled elastic tube is stretched only as long as the internal pressure exceeds the external pressure. These two pressures are exactly equal in the collapsed part of the tube. **C** In a human in an upright position, both arterial and venous pressure at the level of the feet increase relative to the pressure at the level of the heart measured on the brachial artery by about 80 mmHg. When the hand is raised above the head, the blood pressure at the wrist level is approximately 50 mmHg lower than the blood pressure measured at the shoulder of the lowered arm, and the effective venous pressure above the heart is less than zero. Down arrows—the blood flow coincides with the direction of the hydrostatic pressure. Up arrows—the blood flow is against the direction of the hydrostatic pressure

The reality of the significant influence of gravity (the hydrostatic effect) on hemodynamics in humans, as an upright creature, has already been demonstrated [6, 9, 26, 33, 37, 38] (Fig. 2).

**Fig. 3:**
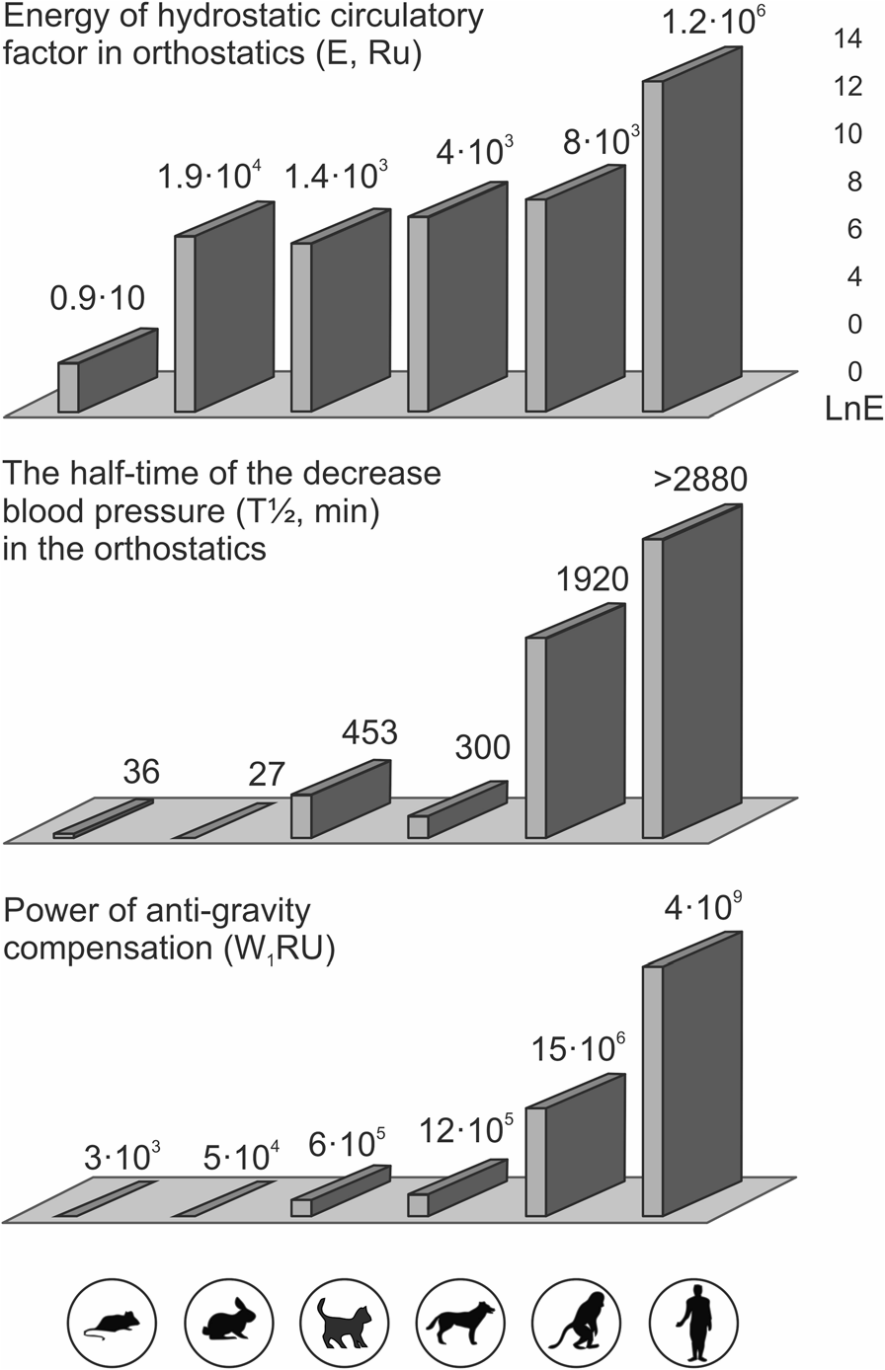
Comparative characteristics of adaptation of the cardiovascular system (in terms of circulatory stability, manifestation, and compensation power) to the influence of gravity on blood circulation according to Belkania et al. [11]. *E* = *p* × *m* × *g* × *h* [RU], where *m* = *V* (blood volume, [ml]) × *p* (blood density, taken as 1), *g* – acceleration of gravity (taken as 1), and *h* – height of the hydrostatic blood column [cm]; T½ [minutes] – half-time of the decrease in blood pressure in orthostatics [minutes]; *W* = *LnE* × *T*½ [RU]

The value of the physical equivalent of the hydrostatic effect (E, Relative Unit – [RU]) in primates, especially in humans with their semi-vertical and vertical postural statics, is several orders of magnitude higher than in pronograde animals (Fig. 3) This was arrived at by calculating the approximate value of the hydrostatic component of blood circulation in orthostatics, taking into account the different values of *m* and *h* for the vascular bed below the heart level in rats, rabbits, cats, dogs, monkeys, and humans. Moreover, the real interspecies differences and the very high degree of adaptation of the CVS to the influence of gravity on blood circulation (in terms of compensation power—W [RU]) in primates, especially in humans, can be observed when comparing the degree of the manifestation of the hydrostatic effect (E) with resistance to orthostatic effects (for the half-life of the decrease in blood pressure – T½ in minutes).

**Fig. 4:**
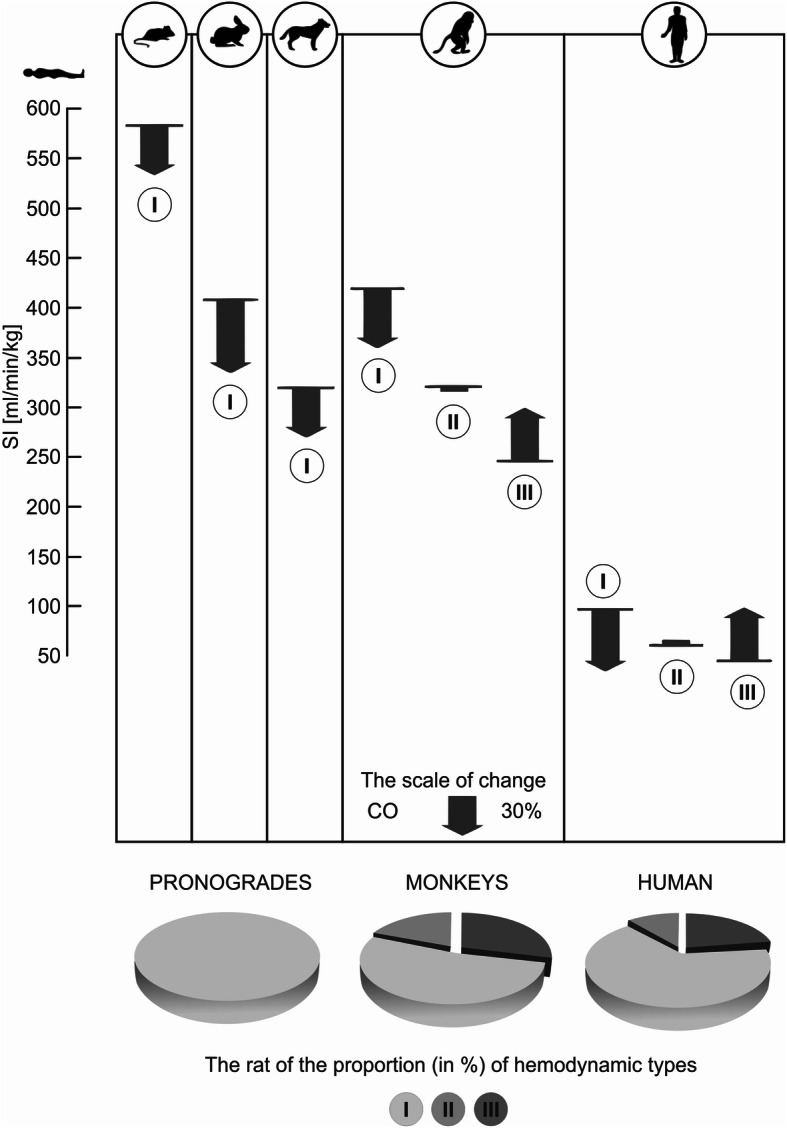
Comparative characteristics of the typological structure of blood circulation in animals with pronograde positional statics and four-legged locomotion (rats, rabbits, dogs etc.) and in primates (monkeys and humans). The prevalence of I, II, and III types of hemodynamic reaction by the ratio of minute blood volume (CO, in %) in standing/lying transition is illustrated. The arrows show the direction and magnitude of changes in CO (in %) in the vertical body position in relation to the value of CO in the horizontal body position in restrained animals and in humans when lying down, taken as 100% (the scale of change is 30%). The profiles of specific types of hemodynamic reaction are presented at levels that correspond to the value of the systolic index (*SI = CO* / *body weight*, [ml/min/kg]) in the horizontal body position/lying down position [11]

For creatures with semi-vertical and vertical statics (such as monkeys and humans), the influence of gravity (hydrostatic effect) on the cardiovascular system is an internal circulatory effector that also determines the dynamic functional organization of the system. The latter manifests itself in clear differences in the ratio of cardiac output (CO) when switching from a vertical to a horizontal position, taking into account the importance of the influence of gravity on blood circulation in primates in contrast with pronograde animals with four-legged locomotion (Fig. 4). The differences in the ratio of CO in response to a change in body position determine the type of cardiovascular response to gravitational forces at rest. Namely, the cardiovascular response with the cardiac output in the upright position below 95% of the cardiac output found in the supine position is classified as type I (hypokinetic). Type II (eukinetic) describes the reaction when cardiac output in the standing position is above 95% and below 105% of that found in the supine position. And type III is recognized when cardiac output in the standing position is more than 105% of the cardiac output found in the supine position [35]. In all pronograde animals with four-legged locomotion (rats, rabbits, dogs, etc.) in a vertical body position (head up, in orthostatic position), cardiac ejection is determined only by one type of hemodynamic reaction—hypokinetic (I), whereas the typological structure of the functional organization of the circulatory system in primates (monkeys and humans) is represented by all three types of hemodynamic reaction—hypokinetic (I) with a decrease in CO in the orthostatic position; eukinetic (II) with no change in cardiac output; and hyperkinetic (III) with an increase in CO in the orthostatic position [29, 31, 34, 35, 39].

A very significant characteristic of types I, II, and III of the hemodynamic reactions in monkeys and humans (other animals do not have types II and III in natural conditions) is a particular correlation between the stroke volume (SV), cardiac output (CO), and total peripheral resistance value (Fig. 4) in the horizontal position [11, 29]. In type I, in comparison with type III, the greatest values of cardiac output (systolic index—SI or CO) and, accordingly, smaller values of indicators of the total peripheral vascular resistance are determined in the horizontal position. Conversely, in type III, the lowest values of cardiac output and the highest values of vascular resistance are determined in the supine position. Type II (eukinetic) is an intermediate type between types I and III. As presented in Fig. 4, the differences in systolic index (SI) in the supine position in monkeys, who have the same three human-like types of structure of the dynamic organization of cardiovascular system regulation, are more pronounced than in humans.

These data suggest that the basic phylogenetic focus of the dynamic organization of the circulation by the influence of gravity in the transition from pronograde animals with quadrupedal locomotion for primates, with their half-upright (monkeys) and vertical (humans) postural statics, is the formation of a hyperkinetic type of hemodynamics with an increase in cardiac output in the upright position, reflecting the anti-gravity potential of the cardiovascular system. The transition to type III is, on the one hand, an adaptive manifestation aimed at maximizing the circulation when walking upright through the cardiac output. On the other hand, this is accompanied by significant restrictions in the reactivity of the CVS, which defines this type as suboptimal [20–22, 31, 35, 38–40].

**Fig. 5:**
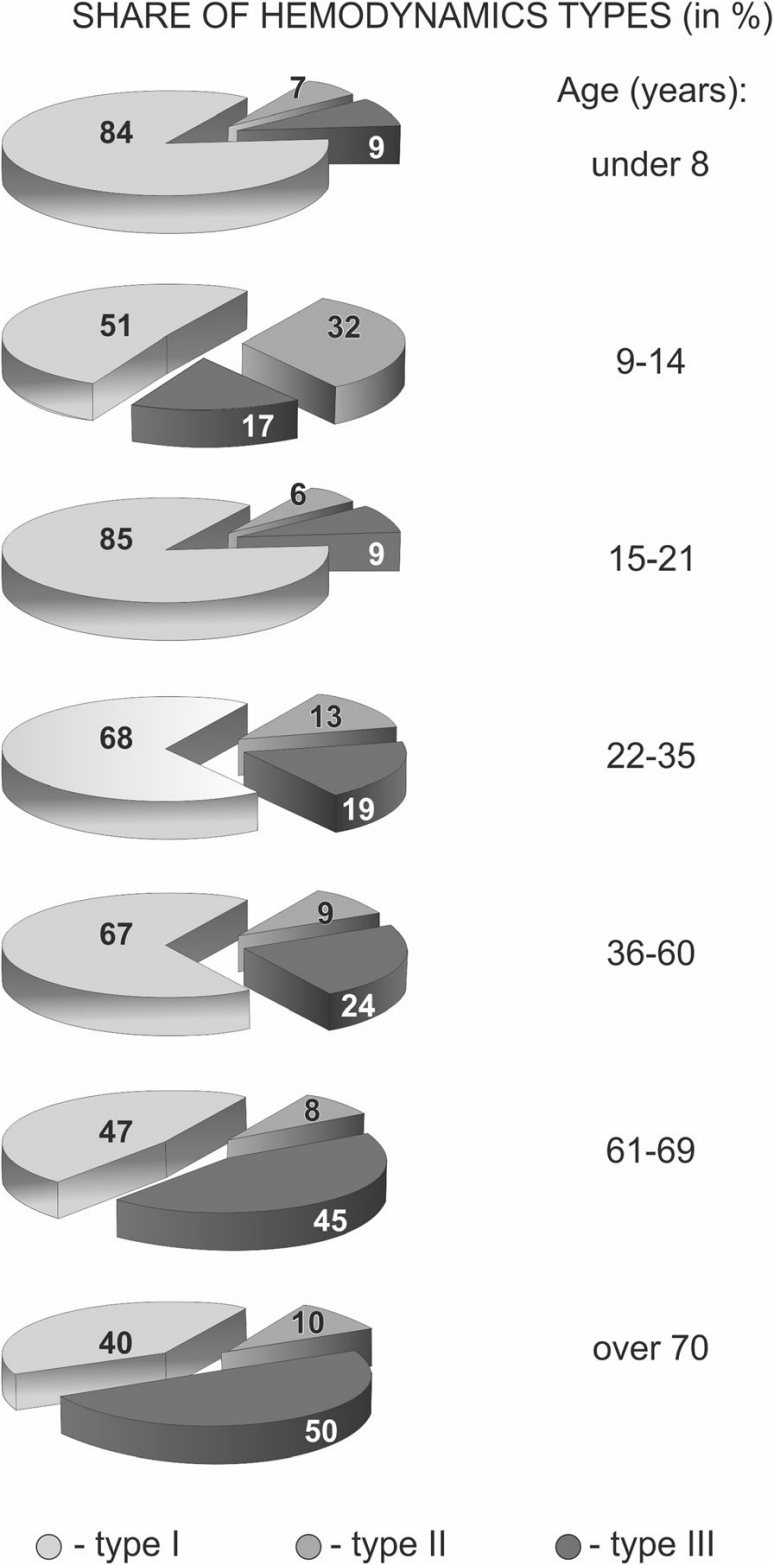
An anthropogenic model of age dynamics of the typological structure of blood circulation in humans based on the ratio of cardiac output (CO, in %) in standing/lying positions [11]

It should also be noted that a similar phylogenetic orientation with an increase in the hyperkinetic state of the CVS (type III) is also determined by the age dynamics and probable age-related transformation of the typological organization of the vascular state in monkeys [11] and in humans (Fig. 5). Figure 5 clearly presents that the share of hyperkinetic (type III) hemodynamic reaction to the change of the influence of gravity clearly increases during the reproductive age and, especially, during the post-reproductive age. At the same time, this transition is enhanced by comorbid conditions, especially by the presence of arterial hypertension in both humans and monkeys, as representatives of the same order of primates [6, 11, 20, 21, 23, 27, 28, 39–42].

**Fig. 6:**
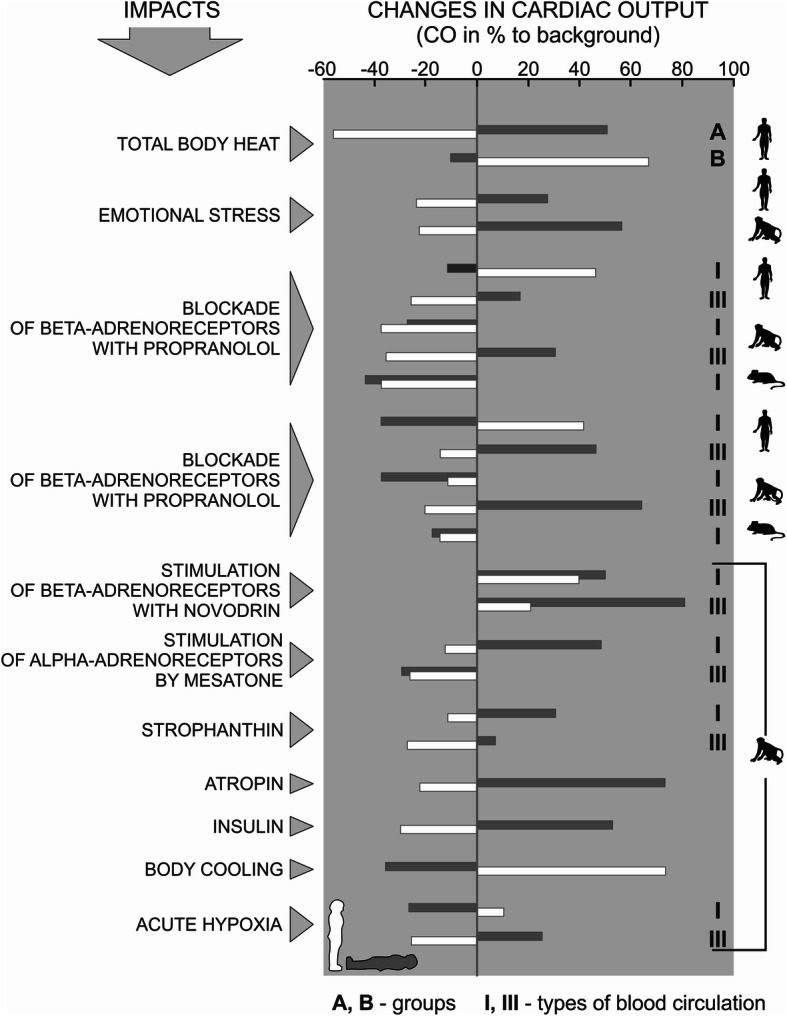
Comparative typological characteristics of the cardiovascular system reactivity (according to changes in cardiac output—CO) in pronograde animals with four-legged locomotion (rats) and primates (monkeys and humans) in types I and III of blood circulation in horizontal (lying—grey bars) and vertical (white bars) body positions [11, 29]

It is important to note that the typological structure determines not only the basic state of the dynamic organization of blood circulation, but also the reactivity of the CVS to various conditions. Figure 6 shows the cardiovascular responses to various stimuli in pronograde animals with four-legged locomotion—rats (only type I hemodynamic regulation) and primates (monkeys and humans) characterized with all three types of hemodynamic reactions (hypokinetic, eukinetic, and hyperkinetic) in the horizontal position (grey bars) and vertical position (white bars). Figure 6 shows how the change of CO can be the opposite for the same impact on changes, both between extreme types (I and III), and for the same type, but in different body positions, lying or standing. An application of the same stimulus in primates (monkeys and humans) can result in the opposite changes in CO depending on body position during stimulation. However, the direction of these changes strictly depends on the type of hemodynamic regulation of the cardiovascular system. Figure 6 presents two extreme types (I and III) during the experiment. Changes of CO in rats representing the pronogrades (Fig. 6) in the horizontal and vertical body positions during exposition to different conditions were unidirectional, which is unlike the primates [11, 29].

**Fig. 7:**
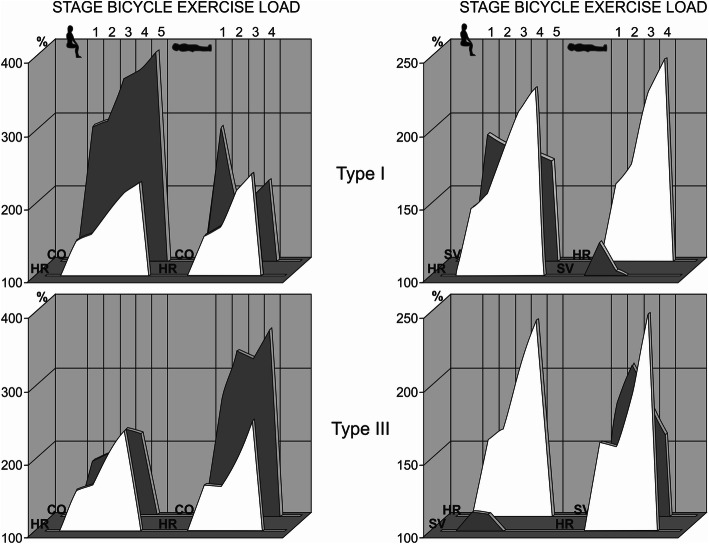
The typological characteristic of the efficiency (the ratio of cardiac output to the heart rate) of hemodynamic support in humans for dosed physical activity with standard veloergometry in the sitting and lying positions (indicated by figures) [35]. The initial values before the load are taken as 100%. Grey profile: *СO* cardiac output, *SV* cardiac stroke volume. White: *HR* heart rate

In Fig. 7, significant typological differences are presented between types I and III of CVS reactivity for cardiac output during dosed exercise (veloergometry) in the sitting and lying positions in healthy humans [29, 31, 35]. These differences are particularly pronounced in terms of the cardiac stroke volume (СSV).

## Energy “costs” of human adaptation to gravitational forces

The exceptional importance of upright walking as the main postural condition of life throughout the age dynamics of human adaptation to the Earth’s gravity and its intensity is emphasized by the energy “cost” of such adaptation.

It is clear that the active counteraction of the body, especially its musculoskeletal system and blood circulation, to gravitational forces requires an appropriate energy supply. In medium-sized land animals, the energy expenditure for compensating environmental conditions in the gravitational field is between 20 and 27% of total energy expenditure.

Larger animals with a body weight of about 70 kg have higher energy consumption which accounts for about 40% of total energy consumption for the same motor activity [43–45]. For humans, the gravitational component of energy consumption is even higher and varies between 40 and 50% of all metabolic energy [33, 46–48], and it is even higher when the body is weakened (by illness, fatigue, malnutrition, etc.). For this reason, the overall level of metabolism in humans throughout postnatal ontogenesis is relatively higher than in other animals.

**Fig. 8:**
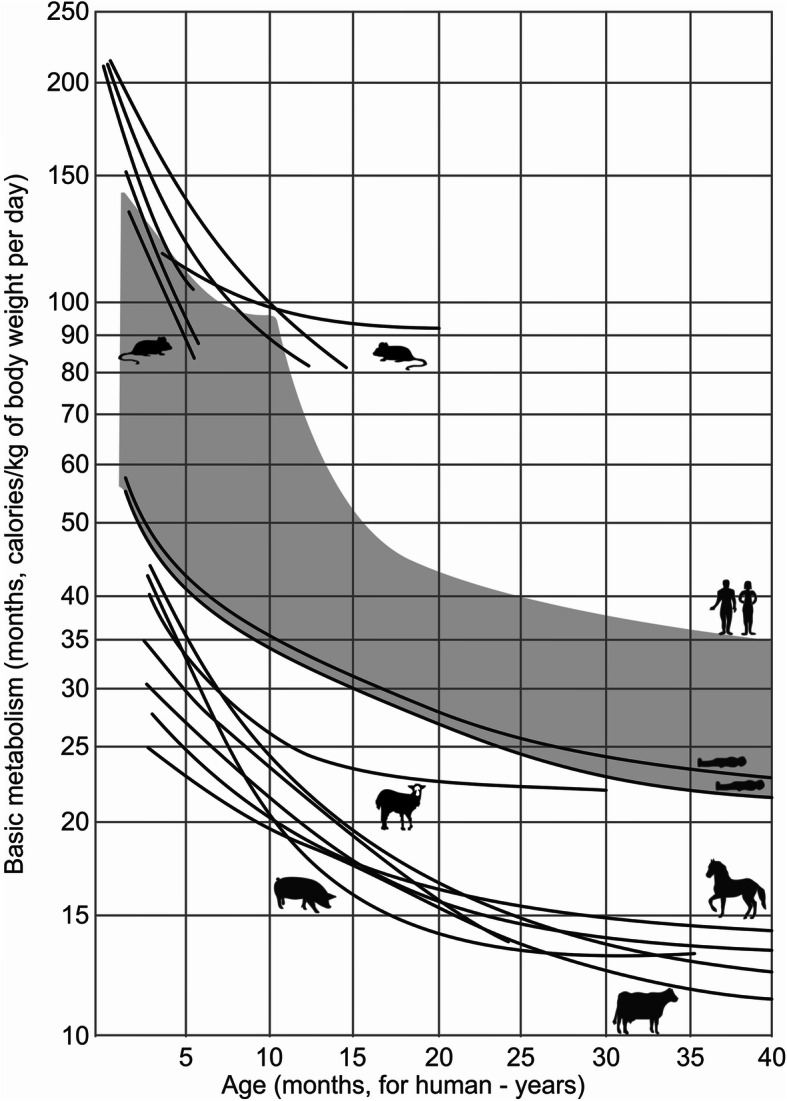
Composition of the fragments of a logarithmic scale of age dependence of metabolism (calories/kg of body weight per day) in small animals (rats—the upper group of curves) and large animals (sheep, pigs, horses, cattle—the lower group of curves). Human data (highlighted in the grey zone) are shown for humans in two positions: the supine position (lower and upper curves, respectively) and standing (the upper edge of the darkened zone in accordance with the increase in energy exchange indicators during standing still in relation to the level of the main exchange [29, 31]

According to the summary data [29, 31] presented in Fig. 8, it can be clearly seen that, in different mammals, the overall level of the basal metabolic rate decreases with increasing size of the animal. In principle, the same pattern appears within each animal species, including humans, throughout ontogenesis—first a rapid and then a slow decrease in the level of metabolism.

It should be noted that even when focusing on the standard conditions for determining the basal metabolic rate in humans in the supine position then, in this case, the overall level of metabolism in humans, as noted above [32, 43, 47, 48], is significantly higher than in animals with a similar body weight. At the same time, it should be borne in mind that, in principle, a condition comparable to four-legged animals for determining the basal metabolic rate in humans is not lying down, but standing up in a relaxed state. Therefore, when comparing the intensity of human metabolism with other animals taking into account the abovementioned conditions (see Fig. 8, the upper edge of the darkened zone), it is quite obvious that a higher level of energy consumption is observed in humans. The higher energy cost of walking upright, as the main postural condition of human life, is revealed by a comparative assessment of oxygen consumption in different sized animals, including humans, for moving one kilogram of body weight for a distance of one kilometer [32, 33, 43–45].

The high energy consumption of upright walking of the human species and the corresponding increase in heat production required the formation of appropriate heat transfer mechanisms which were more effective than those of other animals. Hence the unique human characteristic of skin as the main effector organ of thermoregulatory support and the importance of skin blood flow as an important component of blood circulation. The function of human skin and skin blood flow refers not only to the main circulatory mechanism of thermoregulation, but also to the participation of the redistributive regulation of systemic circulation in ensuring the adaptation of the CVS to the hydrostatic effect of gravity in conditions of upright walking. So in human evolution, second to the development of bipedalism, a rudimentary coat and switching to cutaneous blood flow as the main mechanism of heat transfer sharply increased the intensity of life in the vertical body position. This includes the formation of typical human behavioral thermoregulation forms: primitive clothing; warming at the open hearth; anthropogenic changes in living space at home; and the formation of new energy sources. In other words, first of all, in a dependable animal organism that ensures the life activity of an upright creature in unique conditions of very high energy expenditure, an intensive anti-gravity tension of the basic functions (like motor, circulatory, and thermoregulatory function) is observed in comparison with other animals. The radical modification of the thermoregulation system and the achievement of a high degree of efficiency in upright humans reflect the importance of energy stress and heat stress (external and internal origin) as a pressure factor in the evolutionary selection of hominids [11]. The increasing energy “cost” of adaptation to the Earth’s gravity in conditions of upright walking is determined by this internal factor.

## Changes in the cardiovascular system during postnatal ontogenesis as an anthropogenic basis for the physiology and pathology of the cardiovascular system

Morphological and functional changes in the cardiovascular system in postnatal ontogenesis are primarily associated with growth processes at the pre-definitive stage and subsequent structural, functional, and nosological transformations at the definitive and post-definitive stages of organism development. However, it should be emphasized that this results not only from the parallel growth of organs and the functional formation of the CVS, but also from the hemodynamic support of the growth process as a whole, and in the future of an individual, the vital activity of the body in all forms of manifestations of the somatic state and in the full functional range of “health–illness–pre-illness–disease.”

In the context of age-related dynamics of the formation and function of the CVS, the “ontogenetic model” of the CVS should be considered as a system of basic growth support, physical development, and vital activity of the organism. However, the completeness of such a model should be determined considering the specific features of human biology. The biological quality determines the direction of the stage-by-stage processes of growth and physical development and the specific features of ontogenetic adaptations of the organism. Such an evolutionary conditioned, ontogenetically formed, and fixed quality for a human is being erect. This primarily determines the features of all of the main components of anthropogenesis (morphology, physiology, and psychology) [1–4, 12–14, 28, 29, 32, 43, 49–51]. There is reason to believe that erectness also determines the nosology of a human, determining the specific features of morphological, functional and pathological transformations (pathological anthropology) of the somatic state of the organism during growth, physical development, and life activity in postnatal ontogenesis. This means that the “anthropogenics” in humans also permeates the “ontogenetic,” defining the features of all processes in postnatal ontogenesis.

Therefore, the “anthropogenic model” is considered not only as a step-by-step ontogenetic formation of upright walking, which is the main postural and motor form of human activity as an upright being, but also as a permanent organizational adaptation to Earth’s gravity in all of its physiological and pathological manifestations [3, 4, 6, 8, 29, 37, 49, 52–54].

**Fig. 9:**
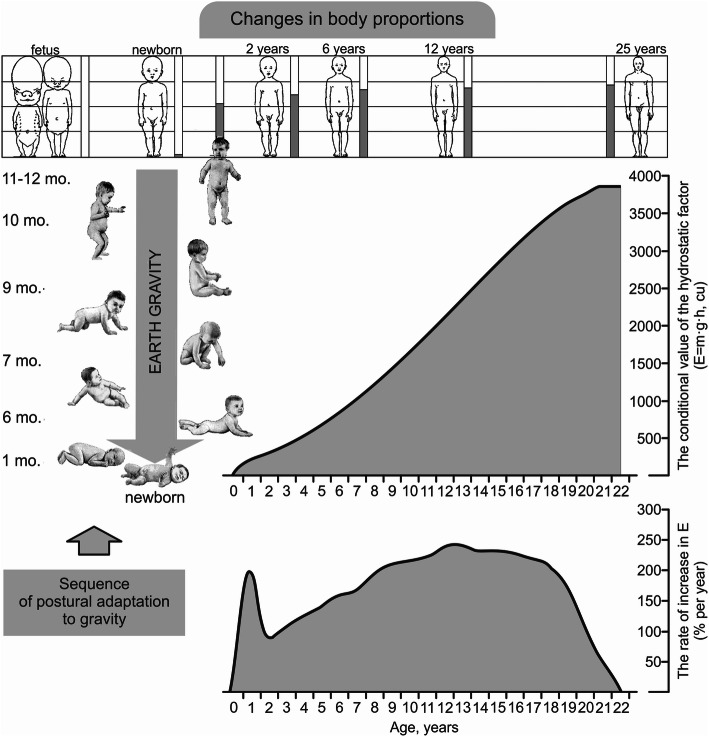
The increase of the hydrostatic (the influence of gravity) effect of blood circulation in the process of growth and gradual transition to erection during the *pre*-*definitive* stage of postnatal ontogenesis. Changes in body proportions are shown in accordance with Robins et al. [55]

This adaptation takes place continually throughout the entire organism growth period (increase in linear dimensions and changes in body proportions) [29, 55, 56], and especially with the gradual transition to erect walking during the first year of life (Fig. 9). As noted above, the hydrostatic column of blood also increases in line with the increase in total length of the body (height—*h*) and body mass (*m*). The relevance of this for blood circulation is enhanced by the formation of the vertical posture and the transition to life in an upright posture (sitting, standing, walking). In Fig. 9, a permanent increase in the height of the hydrostatic blood column is shown by a bar chart (darkened part) next to the body figures. The increase in body weight is accompanied by an increase in blood volume and mass, which increases the weight component of the hydrostatic blood column. In this case, the conditional value of the hydrostatic effect (*g* × *m* × *h*, [RU]) of blood circulation increases by several orders of magnitude (Fig. 9) [57].

On the graph, the rate of change in the value of the hydrostatic effect (Fig. 9) shows that its value (in percentage per year) increases throughout the definitive period of postnatal ontogenesis, up to end of the organism growth. Moreover, the first peak of the rate of “*g* × *m* × *h*” increase is marked by the end of the first year, when the child independently gets on its feet, and the hydrostatic conditions of blood circulation changes fundamentally. Then, for 2–3 years, the rate of increase in “*g* × *m* × *h*” slows down, after which it progressively increases until 12–13 years of age. It remains at a consistently high level until 17–18 years of age, and during the last period of overall growth (21–22 years of age) it progressively decreases to zero.

Thus, the most dynamic periods of the formation of the hydrostatic circulatory effects in a human is the first year of life (transition to upright walking), and then from 4–5 years of age to 12–13 years of age (to the beginning of puberty). This means that in these periods, the most pronounced instability of anti-gravity stress of blood circulation manifests. The regulation of blood circulation according to the hydrostatic effect in the period of puberty (12–18 years of age) remains in a state of stable tension. At the completion of physical development and the end of the growth stage (by 21–22 years of age), the hydrostatic situation of blood circulation and its regulatory support is stabilized at a final formed level, in accordance with the constitution and final dimensions of the body.

However, in the future life of an individual, during the reproductive and socially active period, anti-gravity tension increases relatively, especially during the first reproductive stage (22–35 years of age), due to an increase in the amount of time of the body being in an upright position (sitting, standing, walking). This is due to a violation of the daily rhythm with an increase in the time spent in a vertical position with a shortening of the lying period, including violations of the natural synchronization of the “day-night” biorhythm. The abovementioned factors result in an increase in the body’s tension in the anti-gravity mode [1–4, 6, 29, 32, 35, 39, 49, 51, 58], which actually leads to the development of fatigue syndrome. Several studies have demonstrated an association between chronic fatigue syndrome and orthostatic intolerance, predominantly postural orthostatic tachycardia syndrome [7, 59, 60].

**Fig. 10:**
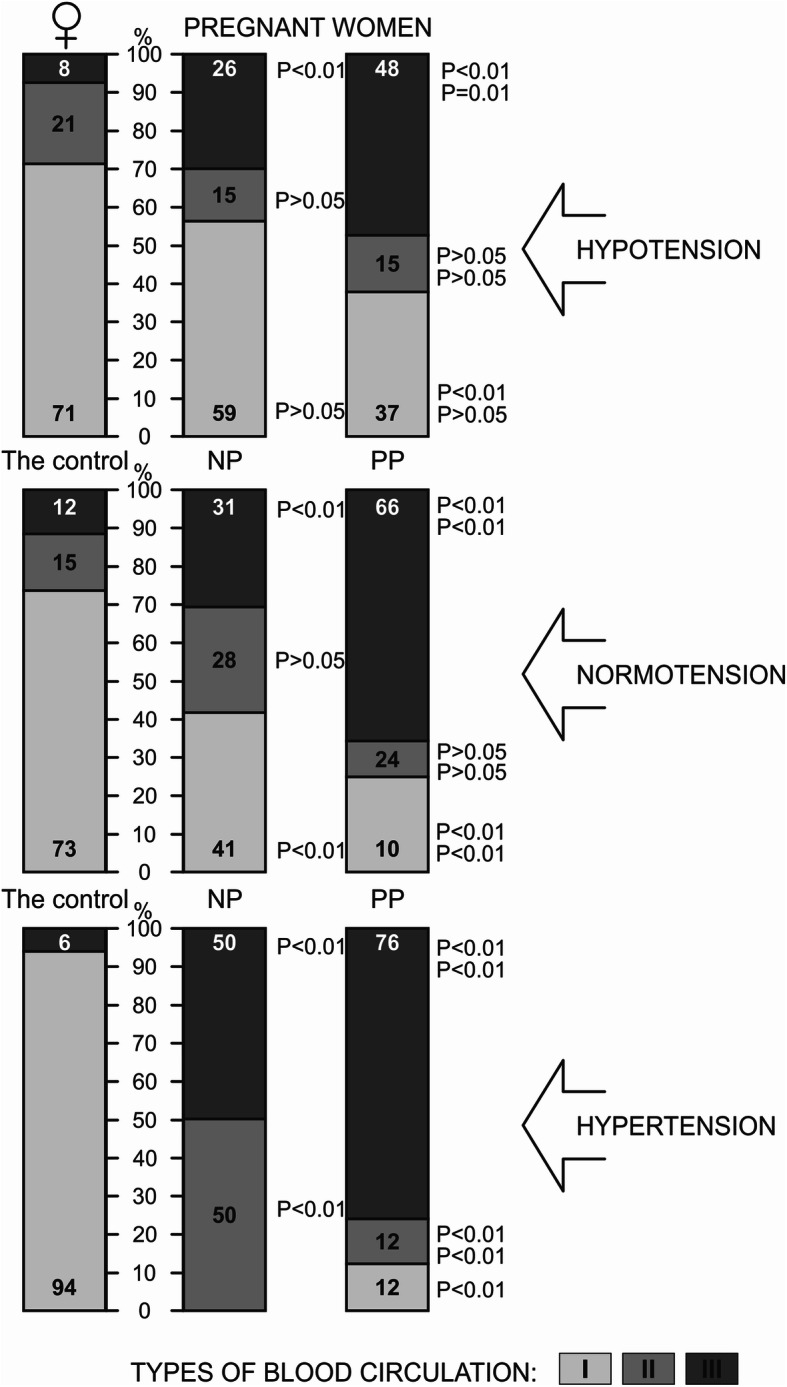
The structure of circulation types (share in %) as the ratio of minute blood volume (MBV) between standing/lying positions [hypokinetic (I), eukinetic (II), and hyperkinetic (III)] for hypotension, normotension, and hypertension in non-pregnant woman (control), in normal (NP) and pathological pregnancy (PP). The significance of the difference (P) in blood circulation types is given between the control and NP, the control and PP (first row), and between NP and PP (second row)

During the reproductive age of women, it is particularly important to note the intensity of the cardiovascular system functioning and load for the influence of gravity (hydrostatic effect) on blood circulation during pregnancy, which is synergistically enhanced by the emerging physical conditions in connection with the process of placentation, growth of the uterus and fetus. At the same time, it is necessary to take into account the changes in the woman’s organism as a form of preparation for long-term pregnancy with a large fetus and, unlike other animals, mainly in an upright position of the body [36, 41, 61–63]. Hence, increased anti-gravity stress, vasoconstrictor regulation in this position of the body can become the basis of preeclampsia and the formation of arterial hypertension in pregnant women [17, 41], all the more that preeclampsia was observed primarily and predominantly in human beings [64]. Predictor of this variant of pregnancy course is a clear increase in the type III of dynamic organization of blood circulation (Fig. 10), especially in arterial hypertension and hemodynamically identifiable heart failure by perfusion type [26–30, 37] or in the pathology of pregnancy [41, 61–63].

Finally, the increase in anti-gravity stress of the cardiovascular system is realized on the background of increasing morbidity during the second reproductive age (over 35 years). Other possibilities of adaptation to the gravitational load when walking upright are determined in humans in the post-reproductive age. It is quite natural that against the background of aging, the ability to maintain the level of anti-gravity stress of the body is significantly weakened. By this time, in addition to painful conditions, a solid “baggage” of the depreciation of tissues and organs, which provided long-term adaptation of the body to gravity, has already accumulated. This explains why more and more people as they get older prefer to be at rest, especially in the supine position.

In this regard, the main non-communicable diseases typical of the human nosological profile are subject to special discussion. These are, first of all, cardiovascular diseases, such as arterial hypertension and hypotension, ischemic heart disease, disorders of cerebral circulation, and insufficiency of arterial and venous blood circulation of the lower extremities. In addition, there also are degenerative diseases of the spine and large joints, primarily the pelvic girdle and lower extremities, stomach and duodenal ulcer, diabetes and a number of other diseases.

On the one hand, against the background of any disease, the tension of the body’s systems in the anti-gravity support mode is further increased. Therefore, a widely practiced medical recommendation for people who are ill is to expand the routine use of bed rest. On the other hand, human diseases are a manifestation of a special (nosological) form of adaptation to the relatively increased influence of the Earth’s gravity [1–4, 29], which leads to disadaptation. This vital anti-gravity stress of the body throughout postnatal ontogenesis, interacting with the so-called risk factors (environmental and organizational origin), determines the anthropogenic basis of the aging process, including the main non-infectious diseases as well as the quality and duration of human life.

It therefore seems appropriate to revise the archaic definition of the term “pathological anthropology,” based on the idea that the development of disease in humans “is not limited to a local process, but affects the entire body” [1, 2, 12, 29]. When considering the intensively developing research in the field of evolutionary medicine, there are quite reasonable arguments to link the definition of “pathological anthropology” with the outstanding biological quality of a human—erect walking [3, 4, 11, 12, 17, 24, 39–41, 50, 57, 58].

**Fig. 11:**
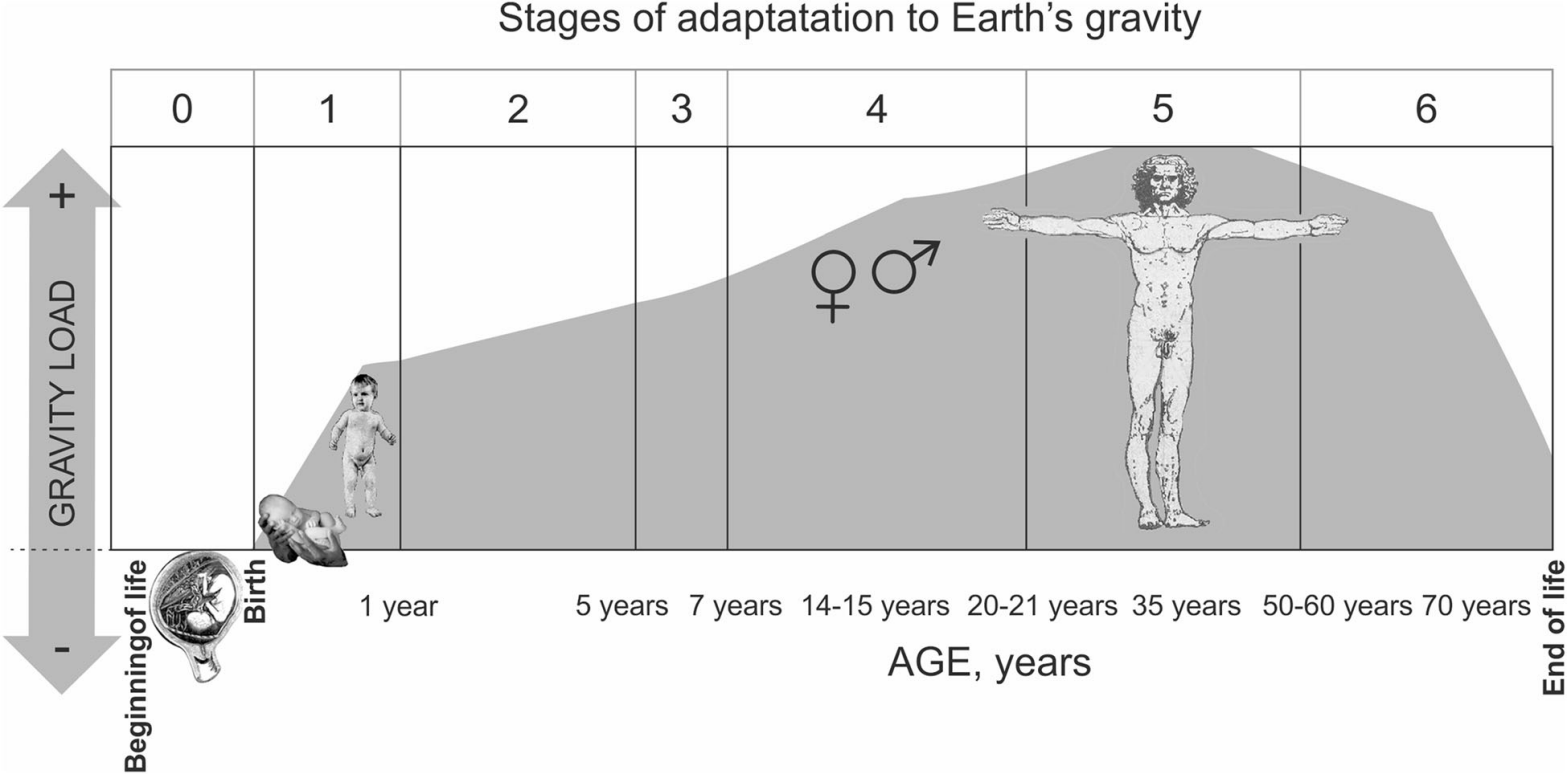
Stages of relative changes of the influence of the Earth’s gravity and organizational adaptation to them in the process of human development and life activity

Figure 11 illustrates the stages of the relative changes in the influence of the Earth’s gravity on the tension of organism adaptation during the process of growth, physical development and human life:

0—prenatal development (from fertilization to birth)

1—the formation of premontane (from birth to 1 year of age)

2—the formation of basic forms of locomotor bipedalism (to 5 years of age)

3—the ability to maintain the body in terms of premontane and bipedalism (to 7 years of age)

4—sexual consolidation of erectness and the formation of semi-differentiated forms of adaptation of the body of women and men to gravity (by 20–21 years of age)

5—reproductive age, including pregnancy in women, and nosological forms of adaptation during the first and second adulthood (from 20 to 21 years of age before menopause in women and up to 60 years of age in men); and 6—aging and amortization forms of adaptation to gravity (after menopause in women and over 60 years of age in men and until the end of life)

In addition to the age limits of the main stages in Fig. 11, the phrases on the age scale indicate the dividing date within specific stages: in stage 4—the phrase “14–15 years” corresponds to entry into puberty; in stage 5—the phrase “35 years” separates the periods of the first and second stages of reproductive age; in stage 6—the phrase “70 years” separates the phase of post-reproductive age before and after changes in the conditional life expectancy. The latter is taken as the conditional average life expectancy at birth of the total population according to WHO (World Health Organization) [53, 65].

The proposed “anthropogenic model” (periodization) of adaptation to the Earth’s gravity in the process of formation in the womb and during human life in specific conditions of bipedalism [6, 11, 12, 32] is projected into all three stages of postnatal human development (pre-definitive, definitive, and post-definitive) and are well-synchronized with the accepted periodization of human physical development.

## Conclusions

The representation and our classification of postnatal human ontogenesis focused on defining biological quality (erect walking). This makes it possible to consider the main manifestations of a human’s life in all range states (health–illness–disease) as permanently flowing due to a lifelong adaptation to the Earth’s gravity. This idea is a necessary synthesizing element within the currently established ideas about the causes and mechanisms of development of the abovementioned major non-infectious diseases associated with aging, the so-called four models of medicine by Dilman [50]. If the ecological, genetic, ontogenetic, and accumulative models postulated by Dilman are considered from an evolutionary point of view then it is biologically justified and necessary to be considered on the basis of the anthropogenic model. This, in our opinion, makes a certain constructive contribution to the formation of the theory of medicine, as well as to the development of the means and methods of valeological and medical (preventive, curative) health support [6, 10, 49–51, 58, 66–69].

## Data Availability

Not Applicable.

## Abbreviations

CO: Cardiac output
CVS: Cardiovascular system
E: Energy of hydrostatic circulatory factor
g: Gravitational constant
h: Height
Ln: Natural logarithm
m: Mass
NP: Normal pregnancy
PP: Pathological pregnancy
RU: Relative units
SI: Systolic index
SV: Stroke volume
T½: Half-life
V: Volume
W: Power of anti-gravity compensation
WHO: World Health Organization
